# Vertebrate and Invertebrate Animal Models for the Study of Down Syndrome

**DOI:** 10.3390/ijms26168092

**Published:** 2025-08-21

**Authors:** Ann-Charlotte Granholm

**Affiliations:** Department of Neurosurgery, University of Colorado Anschutz Medical Campus, Aurora, CO 80045, USA; lotta.granholm@cuanschutz.edu

**Keywords:** Down syndrome, chromosome trisomy, animal models, neurodegeneration, Alzheimer’s disease

## Abstract

Down syndrome (DS) is the most common survivable chromosome trisomy, with an incidence of about 1 in 600–700 births. Consequences of chromosome 21 trisomy include developmental delays, congenital cardiac abnormalities, skeletal abnormalities, and age-related dementia of the Alzheimer’s disease (AD) type. Up to 90% of individuals with DS develop dementia symptoms in their 40s or 50s. Because the biological mechanisms involved in DS-related developmental and age-related pathology are less known, animal models consisting of both lower-order and higher-order animals have been developed. We here review the most pertinent and well-studied DS animal models including models developed in *C. elegans*, *Drosophila*, zebrafish, and mice. Molecular pathways involved in DS morbidity that were discovered in animal models will also be discussed.

## 1. Introduction

Down syndrome (DS) is the most common survivable trisomy, with a complete or partial triplication of human chromosome 21 (Hsa21) [1,2]. This condition leads to developmental delays and intellectual disabilities as well as age-related Alzheimer’s disease (AD). Individuals with DS have a range of co-morbidities that can shorten lifespan, including thyroid dysfunction, congenital heart malformations, and sometimes attention deficit/hyperactivity disorder (ADHD) [3,4] or autism spectrum disorder [5]. While between 80 and 90% of individuals with DS develop dementia symptoms [1], 28% to 54% have thyroid dysfunctions, with hypothyroidism the most common, and around 50% have congenital heart conditions, mostly ventricular or atrial septal defects [6]. In terms of autism spectrum disorders and ADHD, it is estimated that 30–40% of those with DS have these co-morbidities [7]. The most common causes of death in individuals with DS are AD, heart disease, and respiratory infections including pneumonia [8]. Hsa21 contains more than 500 genes, and the triplication of this chromosome leads to complex genetic dysfunctions with significant pathological consequences. It is therefore beneficial to use several animal models to fully explore the multiple consequences of aneuploidy of Hsa21. For example, invertebrates have a simple genomic makeup, which is easy to manipulate, so they are suitable for examining specific gene effects during development or aging. Mice, on the other hand, have a more complex genome but are more suitable for behavioral studies including learning and memory.

Hsa21 is the smallest chromosome and was fully sequenced by an international team of scientists [9]. This was the second human chromosome to be fully sequenced, following chromosome 22. The short arm of the chromosome, Hsa21p, encodes largely repetitive DNA and does therefore not contribute significantly to the DS phenotype. The long arm, Hsa21q, contains about 500 functional genes, of which 164 are protein-coding. It also contains at least 5 microRNAs (miRNAs), including miR-99a, let-7c, miR-125b-2, miR-155, and miR-802. Several of these miRNAs contribute to the inflammatory phenotype of DS and/or aggregation of beta-amyloid (Aβ) associated with the early onset AD phenotype of DS [10,11,12,13,14].

Investigators using invertebrate as well as vertebrate animal models have revealed novel molecular pathways that are regulated by Hsa21, by studying orthologous genes. These models have been used for decades to examine novel drug targets, molecular pathways of disease, and behavioral consequences of gene dysregulation and provide increased scientific rigorwhen biological functions are validated at several different levels [15,16,17,18,19]. However, barriers exist that may hinder the effectiveness of existing animal models as complete representations of DS in humans. DS and other chromosomal variations represent complex genetic changes that affect both behavior and physiology. Thus, complete models of this human condition are difficult to replicate in animal models. Lower-order or higher-order animal models for DS must accommodate studies of both developmental abnormalities, on one hand, and age-related conditions such as hearing loss, vision loss, or AD, on the other hand. Barriers for existing animal DS models include incomplete genetic representation, phenotype variability (animal models may only partially replicate these phenotypes), and differences in timing of development or neurodegeneration between any animal model and the human brain. New animal models include, e.g., humanized transgenes for DS-related AD, but their success in directly translating to human therapies is still debated. AD is primarily known as a human condition, with few animal species naturally developing AD pathology, providing a major barrier in examining the physiological consequences of this condition using animal models. This review will highlight the benefits of both invertebrate and mammalian models for DS and their individual contributions as well as their shortfalls for understanding the underpinnings of DS-related pathology, particularly in the central nervous system (CNS).

## 2. Introduction to Animal Models for DS

### 2.1. Invertebrate and Lower-Order Animal Models for Trisomy 21

**Figure 1:**
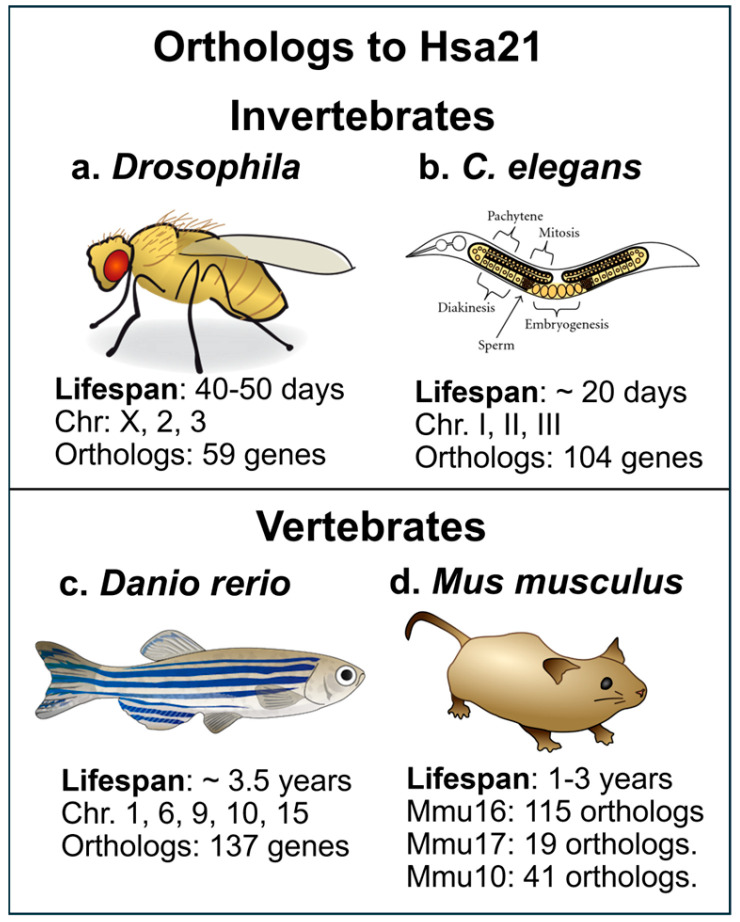
(**a**) The fruit fly, *Drosophila melanogaster*, has a lifespan of 40–50 days and has been used to investigate pathways that are orthologous to Hsa21 changes, for example, the calcineurin pathway; (**b**) The *C. elegans* nematode has also been used for examining specific alterations in molecular pathways caused by Hsa21 trisomy in humans. Developmental and age-related molecular and behavioral changes in genes dispersed over chromosomes can be tested in this invertebrate; (**c**) The zebrafish (*Danio rerio*), a ray-finned fish with translucent fry which are often used in developmental studies, since the entire fish embryo can be imaged and show distribution of dysregulated gene products; (**d**) The mouse, *Mus musculus*, is the most common lab animal and has been easier to manipulate than the rat. The mouse has an average lifespan of 1–3 years, with Hsa21 orthologs dispersed over MMU16, 17, and 10. From: (**a**): https://commons.m.wikimedia.org/wiki/File:Drosophila-drawing.svg (accessed on 2 May 2025); (**b**): https://commons.wikimedia.org/wiki/File:C-elegans-Schematic-drawing-of-the-two-gonads-and-uterus-of-C.jpg (accessed on 2 May 2025); (**c**): https://commons.wikimedia.org/wiki/File:201108_zebrafish.png (accessed on 2 May 2025); (**d**): https://commons.wikimedia.org/wiki/File:Mouse.svg (accessed on 2 May 2025).

Although mouse models have been the most commonly used animal models for DS, lower-order models have their place, due to their relatively simple genetic makeup, shorter lifespan, and easy gene manipulation strategies (Figure 1). As discussed below, these models have unique benefits and drawbacks, but there is no doubt that these lower-order models have contributed to current knowledge, especially in terms of examining gene expression results on specific pathways that are orthologous to the genes on the human Hsa21.

#### 2.1.1. *Drosophila* Models of Trisomy

*Drosophila* fruit flies (*Drosophila melanogaster*) have been used for decades to study the biological effects of genetic manipulations (Figure 1a and [20]). The relatively simple genomics of the fly makes it easy for studies of trisomic conditions. Orthologous genes to the Hsa21 are dispersed on chromosomes X, 2, and 3 in *Drosophila* [16]. Trisomic flies with an extra fourth chromosome survive at rates equivalent to disomic flies and exhibit phenotypic differences, while triplication of the second or third chromosome in *Drosophila* is rarely survivable. Interestingly, *Drosophila* has a built-in dosage compensation. Thus, male and female flies exhibit similar levels of X-linked gene expression, leading to males having a two-fold upregulation of the X chromosome to match the expression levels in females, who have two X chromosomes. It is challenging to create a *Drosophila* model that has a complete or near-complete trisomy of the Hsa21 orthologous genes since the orthologs in the fly genome are dispersed over several chromosomes, as mentioned above. *Drosophila* models have provided valuable information to the field regarding functional consequences of specific molecular pathways altered by chromosomal triplication [19].

Studies have shown similarities in over-expression of certain genes in *Drosophila* that correlate with similar changes in fetal tissue with Hsa21 trisomy. One example of a pathway studied in *Drosophila* is the calcineurin signaling pathway [16], which is also dysregulated in other animal models for DS, such as the Ts65Dn mouse model [21]. The calcineurin pathway is also dysregulated in humans with DS via over-expression of genes on Hsa21 including Regulator of Calcineurin 1 (*RCAN1*) and Down syndrome critical region 1 (*DSCR1*). Over-expression of these two genes leads to suppression of calcineurin activity, resulting in developmental problems. There is a feedback loop where calcineurin regulates the transcription of the DSCR1 protein, providing a fine-tuned regulation of this important pathway. The over-expression of the RCAN1 protein leads to a downregulation of calcineurin signaling in DS and is thought to directly contribute to intellectual disability and learning deficits. Calcineurin inhibitors are immunosuppressants that have been proposed for clinical use in transplantation and autoimmune diseases. Since calcineurin is also implicated in AD, it is possible that calcineurin inhibitors could prevent AD in DS as well [22]. The *Drosophila* model played an important role in revealing the dysfunction of the calcineurin pathway in DS, since the *Nebula* gene in *Drosophila* is an ortholog of the human *DSCR1* gene and is involved in learning and memory in the fly model [16]. The *Drosophila* fly has a lifespan of 40–50 days (Figure 1a), making aging studies relatively simple compared to higher-order animal models. The *Drosophila* genome has been sequenced in its entirety, and this invertebrate was one of the first eukaryotic genomes to be sequenced, providing significant genetic tractability [23]. However, Hsa21 gene orthologs are dispersed in several chromosomes of the *Drosophila* genome, and conserved regions are short (see Figure 1a) as detailed elsewhere [19]. A barrier with the *Drosophila* model is that gene-gene interactions may be better examined with other models and complex cognitive studies cannot be conducted using this model. Fruit flies and humans are evolutionarily distant and therefore display significant differences in molecular function and physiology, lessening the translatability of findings to the human condition.

#### 2.1.2. *C. elegans* Models for Trisomy 21

Similarly to *Drosophila*, the *C. elegans* nematode (*Caenorhabditis elegans*) has a relatively simple genome and a well-defined genetic system, making it easier to manipulate and study than many other animal models [19]. Despite the simplistic genome, many fundamental biological processes, including meiosis and chromosome segregation, are conserved between *C. elegans* and higher organisms, including humans. *C. elegans* can be used to model trisomy, particularly autosomal trisomy. Specifically, *C. elegans* with an extra X chromosome (trisomy for the X chromosome) has been shown to be viable and fertile, contrary to trisomy of the X chromosome in *Drosophila* [24]. Additionally, some studies have explored autosomal trisomy in *C. elegans*, demonstrating that it can be corrected during oocyte meiosis, leading to a higher frequency of euploid offspring than expected. Researchers have used this model to examine the impact of over-expressing or disrupting gene homologs to those on Hsa21. About 40% of human genes associated with disease have worm orthologues (Figure 1b). Nordquist and collaborators studied loss-of-function mutations of 10 different Hsa21 orthologs in *C. elegans* and their importance for neuromuscular function [25]. They found that three of the 10 orthologs to Hsa21 were required for acetylcholine transmitter release, demonstrating the ability to systematically examine loss—or gain-of function for specific Hsa21 genes, and thus being able to dissect which of the 164 protein-encoding genes on Hsa21 that are crucial for normal development or age-related function. *C. elegans* have a lifespan of approximately 20 days (Figure 1b), and the genetic makeup has been sequenced, making this nematode particularly useful for genetic studies and high-throughput genetic screening. *C. elegans* was the first multicellular organism for which the entire genome was sequenced, with several new additions [26]. The genome consists of approximately 100 million base pairs which are organized into six pairs of chromosomes in hermaphrodites or five pairs of autosomes with an XO chromosome in males [27]. Approximately 52% of Hsa21 genes have orthologs in *C. elegans*; about 104 have corresponding genes in *C. elegans*. An example is NCAM2, located on Hsa21. The *C. elegans* ortholog is NCAM1. Both genes are cell adhesion molecules, and the NCAM-1 role in axonal outgrowth has been studied in *C. elegans* [25]. A shortfall with the *C elegans* model is that they lack a circulatory system and organs including kidneys, liver, and lungs, which limit studies of systemic effects in DS as well as interactions between the CNS and peripheral organs.

#### 2.1.3. Zebrafish Models for DS

Zebrafish (*Danio rerio*) models for trisomy have been developed by manipulating genes orthologous to crucial genes on Hsa21 [19]. This is a ray-finned fish, and while it is a vertebrate animal, the genetic makeup is less complex in terms of development and some features compared to higher-order mammals. The zebrafish represents another lower-order animal model that can be used to examine human disorders due to their short lifespan, transparent embryos, and a simple gene makeup, similar to the invertebrate models described above. Zebrafish models have, for example, been used to examine developmental effects of the *DYRK1A* gene [28], which is highly conserved in the zebrafish and plays an important role in deficits occurring in DS in humans [29,30,31]. The zebrafish can live up to 3.5 years in captivity (Figure 1c) but typically live 1–2 years in the wild, and undergo progressive cellular and behavioral senescence similar to the aging process in humans [32]. The age-related neurodegeneration observed in zebrafish includes gradual neuronal loss and therefore represents a valuable model for studies of age-related neurodegeneration [33]. There is anatomical and molecular homology between the zebrafish and mammal neurotransmitter systems, allowing detailed studies of neuropharmacology in this model [33]. The zebrafish genome is also known in its entirety, and was mapped relatively late (in 2013), compared to other models [34]. Zebrafish have approximately 137 genes that are orthologs to the protein-coding genes on Hsa21, and 35 of those have more than one zebrafish ortholog (Figure 1c). These orthologs represent about 77% of the protein-coding genes on Hsa21. The zebrafish genome also contains duplicated genes, some of which are orthologs of Hsa21 genes.

In sum, a significant benefit of the *Drosophila*, *C. elegans*, and zebrafish models is that they have a relatively short lifespan, and therefore can be generated in large numbers, allowing for efficient screening and analysis of phenotypes related to Hsa21 trisomy. Since many cellular functions and pathways are preserved compared to higher-order species, and orthologs to many genes and pathways in humans are known, these are viable models to explore specific pathway dysregulations as well as to test novel treatment paradigms for individuals with DS. As mentioned above, the genome of each of these three lower-order models has been sequenced, allowing sophisticated gene manipulations. Invertebrates do not have a chromosome that is syntenic to Hsa21, the ortholog genes are spread out over several chromosomes, making it more difficult to mimic the DS trisomy in invertebrates or fish.

### 2.2. Mouse Models for DS

#### 2.2.1. Comparison Between Mouse and Human Pathology

**Figure 2:**
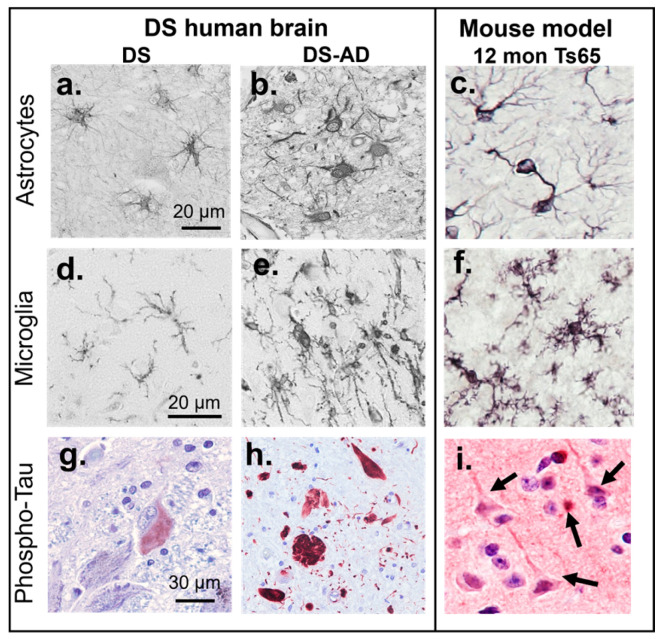
Comparison of pathology in the human brain with DS (**a**,**d**,**g**) or DS-AD (**b**,**e**,**h**) and Ts65Dn mice (**c**,**f**,**i**). Astrocytes in the hippocampus gray matter exhibit a resting morphology in a 25-year-old (YO) with DS without dementia (**a**), compared to the activation and increased immunostaining with antibodies directed against glial fibrillary acidic protein (GFAP) in an individual with DS-AD (**b**) [59] and the increased GFAP staining in hippocampus of a 12-month-old Ts65Dn mouse (**c**) [56]. Microglial activation (stained with Iba1 antibodies) was not observed in a 37-year-old male with DS and no dementia (**d**) but was frequently observed in DS-AD cases (**e**) [59] and in the 12-month-old Ts65Dn mouse (**f**) [56]. The locus coeruleus (LC) in a young (25 YO) individual with DS did not exhibit frank neurofibrillary tangles (NFTs), but a few neurons stained for an oligomeric Tau antibody (TOC1, red chromophore, (**g**) [60]. In DS-AD, frequent AT8-positive tangles and fibers were observed throughout the brain (here a 54-year-old male with DS-AD, red chromophore, (**h**). The mouse brain does not naturally develop NFTs, unless a human mutated tau transgene is introduced, or after injections with human p-Tau (**i**). Tangle-like p-Tau inclusions (see arrows) in large cortical neurons adjacent to an exosome injection from a DS-AD participant’s plasma [61].

Mice have genes that are preserved within mammals [35], but also genes that are not; for example, the human gene for insulin and the mouse gene for insulin evolved from a common ancestral gene. Mouse chromosomes (Mmu) 10, 16, and 17 contain genes that are also found on Hsa21 and are therefore relevant for studying DS. Mmu16 contains the “Down syndrome critical region” (DSCR) including the *Cbr3*, *Dscr1/RCAN1*, *Erg*, *Jam2*, *Pttg1ip*, and *Tiam1* genes [36]. Mice live 1–3 years in captivity but only 12–18 months in the wild (Figure 1d, [37]) allowing a relatively rapid progression of disease phenotype or normal aging. The first mouse model for DS was the Ts65Dn mouse, developed by Muriel Davisson at Jackson Laboratories, and was accomplished by X-ray irradiation of a male mouse [38], with an extra copy of the distal region of the murine Chr. 16, which is partially homologous to the Hsa21. As shown in Figure 2 and in [17,21,39,40,41,42,43,44,45,46,47,48,49], the Ts65Dn mouse develops many molecular and physiological features observed in humans with DS; both during development and aging. The Ts65Dn mice exhibit progressively more neuroinflammation and glial activation with aging (Figure 2c,f, [50,51]), as seen in humans with DS [52,53,54,55], along with age-related loss of basal forebrain cholinergic neurons (BFCNs), locus coeruleus noradrenergic neurons (LC-NE), and subpopulations of hippocampal neurons [56]. Ts65Dn mice have increased APP and Aβ levels and increased intracellular phospho-Tau [57,58], but do not form neurofibrillary tangles (NFTs) or amyloid plaques (Figure 2g,h).

We have demonstrated that the progressive accumulation of p-Tau containing NFTs seen in the human brain with DS-AD (Figure 2h, [62,63]) can be mimicked in DS mouse models by stereotaxic intracranial injection of neuron-derived extracellular vesicles (NDEVs) that contain p-Tau “seeds” from humans with DS-AD (Figure 2i and [61]). Others have shown that exosomes or p-Tau extracted from AD brain tissue also gives rise to the spreading of Tau pathology in the mouse when injected [64,65,66,67,68,69]. This experimental design may represent a new way to induce protein aggregation and AD pathology in DS models, instead of the addition of human transgenes to the mouse genome which could have its own challenges [70]. Challenges with transgenic animals include the potential that the disease phenotype varies depending on the mouse strain background, potential off-target effects, and the risk that species-specific cell functions could affect the interpretation of experimental outcomes. An example of this is that human versus murine microglial cells exhibit different responses to a common trigger despite having many features in common [71]. This could represent a confounding factor. In addition, mice and humans have different chromosomal structures, therefore adding a third copy of Hsa21 to a mouse genome may not replicate the pathophysiology of human DS.

Mouse models are crucial for understanding the role of specific genes and/or gene-gene interactions in the developmental and cognitive deficits associated with DS. In mice, the Mmu16 chromosome contains the largest region of Hsa21 synteny, with approximately 115 orthologs of Hsa21 genes, while the Mmu17 contains around 19 orthologs and the Mmu10 contains roughly 41 orthologs [19]. Sophisticated behavioral and physiological traits could not be studied using lower animals such as the *Drosophila* or *C elegans*.

The most common DS mouse models have a segmental trisomy of overlapping fragments of mouse chromosome 16 (MMU16) that is syntenic to Hsa21. The Ts65Dn model (Ts(1716)65Dn/J) carries an additional mini chromosome with the Mir155 to Zbtb21 region of mouse chromosome 16, homologous to Hsa21. This region includes approximately 90 genes, fused to the centromeric part of mouse chromosome 17 from Pisd-ps2/Scaf8 to Pde10a, which includes 46 genes that are not homologous to Hsa21 and could therefore complicate the interpretation of results in this model, although it was the only mouse model for a long time that recapitulated DS human phenotypes [72], and was therefore used extensively for behavioral, pharmacological, and molecular DS studies [40,47,73]. However, derivations of frozen embryos of the Ts65Dn mouse line have shown significant variability in Ts65Dn lines across generations caused by genetic drifting [38], which reduces the current quality of the model for studying aspects of neurological development in DS. Numerous other models for DS have been developed that may reflect a more accurate genetic representation of Hsa21 (Table 1). There are benefits with having multiple models for DS; this allows us to examine genotype-phenotype relationships by using a combination of transgene and murine trisomy models. We can also identify driver genes in multiple vertebrate and invertebrate animal models and therefore gain rigorous proofs of concept for therapeutics by comparing their effectiveness and potential toxicity in several models. This approach is highly recommended, especially when examining novel biological pathways or novel therapeutic interventions.

Table 1 above shows a summary of the currently developed mouse models and a summarized genetic over-expression and/or chromosomal trisomy theyentail [72,83] and benefits of each model. The specific genes involved in each mouse model have been described elsewhere [19]. With so many DS models it may be difficult to determine which strain to use for a specific experimental question, and this may be limited by the availability of some DS models as well as a lack of studies that demonstrate age-related cognitive dysfunction and neuronal loss in some of the newer models. Although the Ts65Dn mouse described in detail above is known to exhibit age-related learning and memory deficits [40,45,49,84], other DS mouse models may not show these impairments. Thus, the variability of cognitive and neurodegeneration phenotypes in these different models may limit their use for drug therapy or mechanisms associated with cognitive loss with aging, even though they are valuable for mechanistic studies. For example, the adult Dp16 mouse shows cognitive deficits and cholinergic cell loss but lacks the prenatal brain defects seen in humans with DS and in other DS mouse models [75] and, thus, does not fully recapitulate DS in humans.

#### 2.2.2. Comparisons Between Different DS Mouse Models

Several research groups have performed comparison studies of the phenotypes between different DS models, to determine which model is best suited for their studies. An interesting study by Arima-Yoshida et al. [81] showed significant differences between the Ts1Cje, Ts2Cje, and Ts1Rhr mice (see Table 1) in terms of electrophysiological parameters of GABAb receptor function during tetanic stimulation. The Ts2Cje mouse has a slightly larger segmental trisomy of MMU16 compared to the Ts1Cje mice, leading to more severe learning deficits and BFCN degeneration compared to Ts1Cje [85]. This research group was able to demonstrate that a dysfunction caused by the *Carbonyl Reductase 1* (*Cbr1*) gene could be rectified when one copy of the *Cbr1* gene was deleted (Ts1Cje; Cbr1+/+/−), demonstrating the usefulness of studying several models in parallel and combining the trisomy models with targeted gene deletions. These studies demonstrated the importance of correcting the *Cbr1* over-expression which can lead to low blood pressure and regulate sympathetic tone. New druggable targets for humans with DS can come out of this research. Low blood pressure in DS affects exercise tolerance and quality of life, and therefore correction of over-expression of this gene can have meaningful translational effects. Another research group examined differences between the Ts65Dn mouse and the Ts1Cje model showing that the lack of age-related degeneration of BFCNs in the latter model was due to the shorter triplication of MMU16 in Ts1Cje mice, excluding the *APP* gene and demonstrating therefore that APP and Aβ contribute to cholinergic loss in the Ts65Dn mice and most likely also in humans with DS [86].

Reeves et al. [80] developed the Ts1Rhr and Ms1Rhr mouse models that are trisomic and monosomic, respectively, for a region on MMU16 that is homologous to 5.3 Mb of Hsa21; the Down syndrome critical region (DSCR). Utilizing these two models, they found that trisomy for the DSCR alone is not sufficient to produce the structural and functional features of hippocampal impairment that were previously described in the Ts65Dn mouse and in humans with DS. On the contrary, when the DSCR was returned to normal gene dosage (Ms1Rhr/Ts65Dn mice, see Table 1), the performance in spatial reference memory tasks was normalized, thus proposing that the DSCR was critical at least for spatial reference learning and memory. They concluded that even if the critical region hypothesis was disproven by these experimental mouse models, they helped define the contribution of this gene region of Hsa21 and its influence on learning and memory [80].

In a study by Siegel et al. [78], they examined three different DS mouse models for visual discrimination learning as well as inhibitory control. The models were the Dp(16)1/Yey, Ts65Dn, and Ts1Cje models (Table 1). They found that the Dp(16)1/Yey and Ts1Cje models lacked learning deficits during early pre-training, while the Ts1Cje mice had significant learning delays in the late pre-training session, which would suggest frontal cortex pathology [78]. One of the most important findings with this study was that the mouse background strains (which were different between the mouse lines) had significant effects on behavior, pointing out that the background strain is very important when performing behavioral studies and can cause confounding effects. This manuscript points out the importance of selecting the best model depending on which cognitive tasks are proposed and whether developmental or age-related deficits will be examined. Genetic drifting and different genetic background strains can affect the interpretation of morphological, physiological, or behavioral outcomes, since different mouse strains have distinct behavioral traits also when it comes to specific reactions to an introduced mutation or transgene [87,88]. These are examples of the usefulness of studying several DS models in parallel to explore gene-specific influences on DS developmental and age-related phenotypic pathological traits.

#### 2.2.3. The Latest DS Mouse Models

The Ts66Yah mouse model [72] is based on the existing Ts65Dn model and was generated with CRISPR/Cas9 technology [72] removing a specific region of mouse chromosome 17 (Mmu17) on the additional mini-chromosome that is not homologous to the Hsa21 genome. The Ts65Dn mouse carries a freely segregating mini chromosome that includes 13.4 Mb of distal Mmu16 which is fused with 10 Mb of the centromeric part of Mmu17. The Ts66Yah Mouse only has an extra copy of the Mir155 to Zbtb21 region of Mmu16, while the Mmu17 portion is removed. It was created by the Duchon/Herault research groups and was first described in 2022 [72]. CRISPR/Cas9 technology was used to delete a specific 6.2 Mb region of DNA on the mini chromosome, removing genes not homologous to Hsa21. The deletion was performed in vivo, with sperm from fertile males of the Ts65Dn ‘1924’ line and WT F1B6C3B oocytes. One founder carrying the recombined mini chromosome, with the deletion of the centromeric part of Mmu17, was selected and crossed with C57BL/6NCrl females to start this new cohort of mice. It is generally thought that this version of Ts65Dn mice have a milder cognitive phenotype than the Ts65Dn mouse, and that they do not exhibit age-related neuronal loss as some of the other models. On the other hand, this model may be truer to the genes involved in the Hsa21 trisomy. This mouse model presents us with an important conundrum: if a more specific representation of orthologs to Hsa21 protein-encoding genes is generated, using, e.g., the CRISPR/Cas9 methods in the Ts66Yah mouse, and this leads to a milder phenotype with only mild learning and memory impairment and no BFCN or other neuronal loss, where does that leave us? It suggests that the severe learning deficits often seen in individuals with DS, and age-related BFCN loss and cognitive impairment associated with AD pathology cannot be accurately capitulated with a limited specific trisomy of syntenic genes in the mouse. Maybe this is because the mouse is using additional or different genes and/or gene-gene interactions to accomplish the sophisticated learning and memory tasks that scientists have designed. The question is whether it is most important to have a model that does have severe neurodegeneration and cognitive loss or if it is more important to have a genetically accurate model.

The TcMAC21 mouse model of DS was first described in 2020 by Kazuki, Gao, and Reeves et al. [82]. This model was created in a different manner, by incorporating a nearly complete copy of the long arm of Hsa21 (Hsa21q) into a mouse artificial chromosome (MAC); hence the TcMAC21 name [82]. They used microneedle-mediated chromosome transfer, injecting embryonic stem cells, which were then used to generate chimeric mice, where the MAC contributed to brain development. These mice, although less studied than, e.g., the Ts65Dn mouse, exhibit many phenotypes of DS including growth retardation, heart abnormalities, skeletal abnormalities, and neuronal developmental delays [82]. However, studies have not yet been completed regarding the developmental or aging phenotype of the TcMAC21 mice yet (see Table 1). There is hope that the TcMAC21 mouse model will provide a closer genetic model for DS. It has already been shown that TcMAC21 mice undergo age-related BFCN loss (as do the Ts65Dn mice, see above), learning and memory deficits, and elevated levels of APP and its cleavage products, Aβ40 and Aβ42, which contribute to the BFCN degeneration [82]. Cardiac septal defects were found in many of the fetuses, along with cerebellar hypoplasia, and abnormal craniofacial features. Future studies will reveal whether this is the most accurate DS model.

In sum, there have been comprehensive reviews of the mouse models for DS (see, e.g., [1,36]). Therefore, the current review is intended to provide an overview of the most common and available DS mouse models and their basic phenotypes and is not a complete list of all mouse models for DS.

### 2.3. Deficits in Specific Pathways Identified and Examined in Animal Models

Several major pathways that are implicated in DS in humans have been successfully mimicked and studied in lower-order animals including *C. elegans*, zebrafish, and *Drosophila* as well as mice. These include the MAP kinase pathway, the TGFβ pathway, and the JAK/STAT pathway (which led to a clinical trial in DS, see below). Below is a summary of some of the most important pathways that have been examined using the animal models described in this review.

**Figure 3:**
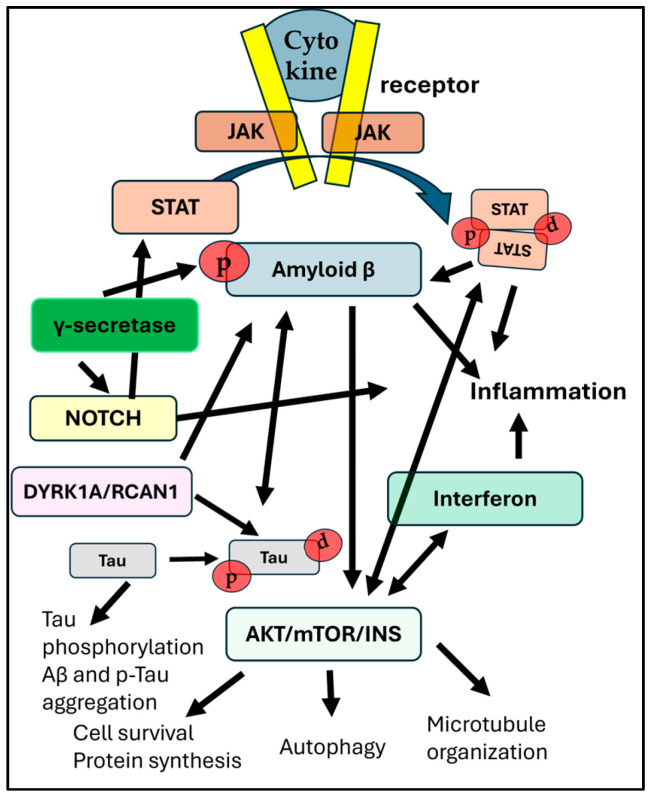
The complex interaction between pathways that are affected by trisomy 21. Arrows signify pathway cross-talks or one-directional influences between distinct pathways. The JAK/STAT pathway is activated by cytokine binding to the receptor and can influence the expression and activity of BACE1 (β-secretase), which is involved in the amyloidogenic pathway. mTOR hyperphosphorylation is linked to increased p-Tau aggregation. Both Notch and APP are processed by γ-secretase. While γ-secretase inhibitors can reduce Aβ production, they also inhibit Notch signaling, which can negatively influence important cellular processes. The interferon (IFN) and Notch pathways interact to influence immune response. The over-production of interferon receptors in DS may have consequences for cell survival and growth factor function, thus interacting also with the mTORC1 or mTORC2 complexes. Dyrk1A and RCAN 1 (both located on Hsa21) can affect amyloid production by phosphorylating APP and are also involved in hyperphosphorylation of tau. Finally, the Notch signaling pathway is involved in neuroinflammation, influencing microglia activation, cytokine release, and the integrity of the BBB. P-tau can promote amyloid-beta (Aβ) production, and Aβ, in turn, can drive tau phosphorylation. Modulating the JAK/STAT pathway is explored as a potential therapeutic strategy for AD, aiming to reduce neuroinflammation, restore neuronal function, and potentially impact amyloid pathology.

- The APP/Aβ pathway plays a crucial role in early onset AD in individuals with DS (see, e.g., [52]). While some of the DS mouse models have elevated levels of APP and/or Aβ, transgenes for Aβ have been used in both *C. elegans* [89] and *Drosophila* models to further characterize their physiological and molecular role in the brain. In *C. elegans*, a transgene expression of human Aβ mutations gives rise to behavioral deficits and shortened lifespans [90], mimicking the effects of this toxic product of APP in humans with DS. Although lower-order animals are suitable tools for screening approaches in terms of anti-amyloid treatments, the molecular effects of Aβ on neurons could not be elucidated utilizing these strains, at least not initially [90]. A series of elegant studies using Ts1Cje, Ts2Cje, and Ts65Dn mice showed that APP is necessary but not sufficient to give rise to age-related cognitive impairment and BFCN degeneration [86], most likely via an amyloid disruption of nerve growth factor mechanisms by binding to NGF receptors [91].
- The Akt/mTOR/Insulin pathway shows an aberrant activation, leading to dysregulation of downstream important pathways in the hippocampus of Ts1Cje mice [79], as well as in Ts65Dn mice and humans with DS [58]. Further, studies in *Drosophila* have shown that increased activity of the mTOR pathway can lead to neurodevelopmental defects and accumulation of AD pathology [92], which mimics aberrant signaling in this pathway in individuals with DS. Both insulin and mTOR signaling pathways are dysregulated early in life in individuals with DS [92,93]. The mTORC1/insulin signaling pathways are key regulators of cell growth and proliferation [94]; this has been shown in multiple animal models. Dysregulation of mTOR and associated signaling can affect autophagy and oxidative stress, which are known to contribute to AD pathology (see, e.g., [87]). In *Drosophila*, the mTORC1 pathway is involved in organ size control and longevity. Patterson and collaborators administered Rapamycin, an mTOR inhibitor, to Ts65Dn mice, giving rise to increased lifespan and health span [95], which was corroborated by several other research groups [15,58]. Thus, the mTOR/Akt/Insulin pathways are highly conserved between different animal models, and the clinical field has benefited significantly from these animal studies.
- The JAK/STAT pathway. The JAK/STAT pathway has been extensively investigated in different DS models, especially as it relates to brain development and to leukemia [96], which has an increased incidence in children with DS [97]. In 1992, the laboratories of Darnell, Kerr, and Stark discovered the JAK/STAT signaling pathway when exploring the cellular response to interferon [98]. JAK/STAT signaling is implicated in neuroinflammation and activation of astrocytes, making it a potential target for therapeutic interventions in the chronically hyper-inflamed DS brain. The connection between impaired JAK/STAT signaling and cognitive function was first described in mouse models, where the JAK/STAT pathway was examined using traditional gene knockout studies [98]. Mechanistic studies of small molecules that can act as inhibitors in this pathway have been carried out in several animal models and have led to clinical trials using the JAK inhibitor tofacitinib, to assess its effects on individuals with DS [99]. Hyperactivation of the interferon pathway and downstream JAK/STAT signaling has been linked to developmental and cognitive deficits [100,101,102].
- The DYRK1A pathway. DYRK1A is a kinase located in the DSCR and is therefore over-expressed in DS. In *Drosophila*, the homolog of DYRK1A is Mini brain (mnb), which is involved in neurogenesis and migration of neurons. By studying the mnb pathway in *Drosophila*, researchers have gained insights into the role of DYRK1A in DS neurodevelopment. Calcium signaling, which is crucial for angiogenesis, has been shown to be dysregulated in DYRK1A-deficient zebrafish, mimicking vascular defects seen in DS in humans [103]. On the other hand, over-expression of DYRK1A in zebrafish leads to enhanced Wnt signaling and inhibited TGFβ signaling [28,104]; similar to the imbalance in these two pathways seen in neuronal progenitors in humans with DS [105]. By suppressing calcineurin signaling in zebrafish and then co-treating with DYRK1A inhibitors, the potential therapeutic benefits and risks with these interventions were studied in zebrafish [104]. DYRK1A over-expression has also been studied heavily in mouse models, leading to the development of inhibitors that can be used for clinical prevention of cognitive deficits in humans with DS. The DYRK1A inhibitor Leucettinib is currently in Phase 1 clinical trial for both AD and DS [106].
- The Notch pathway. Notch signaling is crucial during development, involved in cell fate determination, neurogenesis, and tissue organization [107]. Altered Notch signaling has been implicated in DS, especially during development (Figure 3). For example, dysregulation of the Notch pathway has been proposed to be involved in the gliogenic shift observed in the DS brain [108]. Interestingly, DYRK1A (which is located on Hsa21 and over-expressed in the DS brain) is co-expressed with Notch and over-expression of DYRK1A (as in the DS brain) leads to inhibition of Notch signaling [107,109], altering the fate of neural progenitor cell proliferation as well as neuronal migration that could cause some of the developmental delays seen in children with DS [110]. Studies in *Drosophila* have been instrumental in understanding the mechanisms of Notch signaling and its role in developmental processes, and continued studies using the *Drosophila* model have unearthed new findings regarding this important pathway for neural development [109,110,111], see also Figure 3.

## 3. Discussion

As discussed in this review, animal models have significant value both for discovery of new pathways involved in pathology and for testing of new treatment avenues for comorbidities associated with DS. These could be pathways involved in development as well as aging. Above, we provided examples of crucial pathways that were discovered and studied in animal models first, followed by confirmatory studies in humans. One of the first genes on Hsa21 that was found to be important for the DS phenotype was the DYRK1A gene [20,29,30,104,106,109]. Diligent work by several research groups has now led to a clinical trial with the DYRK1A inhibitor Leucettinib-21 (see above). Although we now have a complete map of the protein-encoding genes on this chromosome, the molecular mechanisms involved in the downstream actions of many long non-coding RNAs and miRNAs located on Hsa21 are still not known. Work remains to be carried out—hopefully including all levels of animal models as well as human tissue—before we have a complete understanding of the developmental and age-related impairments in the DS brain.

Some mouse models for DS have pathological features in common with the human DS brain, while other pathophysiological traits are not developing in the murine system to the same extent as in humans. For example, mice do not develop neurofibrillary tangles (NFTs), nor do they exhibit extracellular amyloid plaques, like those seen in the human brain with AD or DS-AD unless a human transgene is introduced. It is thought that this is due to differences in the murine and human amyloid-beta (Aβ) protein sequence [112]; the three amino acid substitutions in the mouse Aβ sequence are believed to prevent the formation of Aβ plaques in the natural aging process. For the development of NFTs, a crucial component of AD pathology in humans, it is well-known that mice have a different version of the MAPT gene (microtubule-associated protein tau), encoding the tau protein. In humans, the MAPT gene can be spliced into different isoforms, including 3R and 4R, while adult mice only express the 4R isoform. Species-specific differences in phosphorylation of tau in the mouse vs. human brain are also involved [113]. This genetic difference in tau structure affects the tendency of the Tau protein to misfold and aggregate into NFTs [114]. Although these differences may defer studies of protein aggregation in murine models, they still exhibit over-expression of Aβ and some p-Tau isoforms; at least in some mouse strains including the Ts65Ds and the Dp(16)1Yey/+ mouse models [58].

Interestingly, normalizing APP gene copy number in Ts65Dn mice fails to rescue plasticity in this model but can restore the integrity of the basal forebrain cholinergic system [81,86]. Another interesting way to utilize the different models is to differentiate behavioral or morphological differences between them to distinguish the importance of specific genes for DS-related phenotypic changes. For example, Ts65Dn mice demonstrate an abnormality in olfactory system connectivity, but Ts1Rhr mice do not exhibit the same deficits, suggesting that distinct genes or sets of genes that are different between these two strains of mice underlie visual and olfactory system phenotypes, according to William et al. [115]. Buck and collaborators [116] showed that restoring the RCAN1 gene to two copies in the Dp(16)1Yey/+ (Dp16) mouse model for DS reduced wheel running activity and rhythmicity in both light-entrained and free-running young Dp16 mice. Critically, these diurnal and circadian deficits were rescued in part or entirely by reducing RCAN1 to two copies in Dp16 mice, see Buck et al., [116]. RCAN1 and DYRK1A, both genes located on Hsa21, act in concert to regulate signaling pathways that contribute to DS pathophysiology. DYRK1A over-expression phosphorylates RCAN1, which in turn inhibits the calcineurin pathway which downstream can lead to an increased phosphorylation of Tau [117]. Thus, gene-gene interactions are important to examine, and not only the genes located on Hsa21; there are of course cross-chromosomal interactions that are yet unknown.

Despite significant efforts to model DS and/or AD in various animal models, there are methodological limitations and barriers in translating findings to human health and disease. As mentioned above, neither neurofibrillary tangles nor amyloid plaques can be appropriately modeled in mice unless human transgenes are introduced. The transgenic methods are not without problems and can introduce limitations [118]. Overexpression of human APP or components of the gamma-secretase complex including presenilin transgenes can lead to unphysiological interactions with cellular proteins, which can skew results and reduce translatability. Indeed, many proposed AD drugs that look promising in transgenic AD models have failed to show efficacy in humans with AD or DS-AD [119]. These failures illustrate the difficulty in translating findings in animal models to human medicine. Examples of these failures include BACE1 inhibitors, γ-secretase inhibitors, as well as immunotherapies targeting amyloid oligomers or fibrils. Indeed, Aβ antibodies including aducanumab demonstrated significant effects in transgenic AD models [120], but have modest cognitive effects in humans, with significant potentially dangerous side effects. Reasons include that mouse models may not exhibit the entire spectrum of human AD pathology or due to different lifespans of mice and humans, such that temporal components of the disease progression cannot be mimicked in mice. Other potential problems with translation include that some drugs may not cross the BBB in humans, even though they do so in the mouse brain. Finally, AD is a very heterogenous disease including etiology and progression; this cannot be mimicked in mice which are often inbred and of close to identical genetic origin.

In sum, the different DS animal models may exhibit significant differences in terms of developmental or age-related phenotypes and can therefore be used to determine which specific genes and/or proteins that are sufficient to produce cognitive or physiological deficits associated with the DS pathological phenotype in humans. Lower-order animals such as those mentioned here have their specific value, for example the relatively simple genome and the shorter lifespan, allowing rapid and high-throughput studies, while mouse models represent a relatively intelligent mammal that can perform complicated behavioral tasks that are more congruent with human cognitive behavior [121]. These findings show that trisomy manifests as a highly specific modification of the transcriptome within distinct cell types in the brain. All the different models are needed and complement each other in terms of the search for pathophysiological mechanisms as well as therapeutics. Future goals of the DS research community should be developed to improve the utilization of models and to develop additional models that more accurately model human conditions associated with DS and AD. A better understanding of all aspects of brain deficits during development in DS, as well as understanding different facets of AD pathology would improve the usage of correct models for the question at hand. Studies of human brain in DS during development and aging were difficult to undertake previously due to a lack of well-characterized human brain tissues. However, this has been remedied with the development of brain repositories focused on DS [57] and will now allow important parallel studies of human vs. mouse DS-related pathologies.

## 4. Conclusions

Down syndrome is a complex condition that leads to both developmental and age-related deficits and the phenotype of this condition is often complicated by multiple possible comorbidities [1,29,50,52]. The modeling of DS in lower-order animals and mammals has provided unprecedented benefits for individuals with DS, both in terms of understanding the pathophysiological effects of trisomy 21 and for testing a myriad of different therapeutics. As the models have evolved, they have unearthed multiple important biological pathways that can be targeted for successful intervention; both to minimize developmental issues and for combating age-related dementia and AD pathology in individuals with DS. Due to species-specific variations in gene expression and posttranslational modifications (PMTs), a complete translation of findings in animal models to the human condition cannot be obtained, and treatments that were effective in these models have not always translated well to humans. This is because the chromosomes that make up the genomes do not align across species.

The DS population has tripled their lifespan just during the last 30 years, due to modern medicine and heightened knowledge regarding cardiac malformations and metabolic dysregulations. Because of this increased lifespan, it has become evident that most individuals with DS will develop AD pathology and dementia if they live long enough. Future studies need to consider species-specific protein function to design appropriate interventions. It is still debated in the field which model is more suitable for specific studies and depends on the scope of work as well as investment in previous models. Many scientists recommend using a couple of different models in parallel experiments to perform thorough studies, but this is not always feasible due to access or costs. While the Ts65Dn mouse model is most widely used and was recommended by the NIH, new models highlighted here (such as the Ts66Yah model) might be more accurate when appropriately characterized.

## 5. Summarization in Molecular Aspects

This review covered only a small number of the many pathways that are likely involved in development and aging of the DS brain. The following molecular pathways involved in DS pathology were discussed, as well as interactions between them:

- The APP/Aβ pathway;
- The Akt/mTOR/Insulin pathway;
- The JAK/STAT pathway;
- The DYRK1A pathway;
- The Notch pathway.

## Figures and Tables

**Table 1:** Summary of mouse models for Down syndrome. Each of these models has specific advantages based on the purpose of the study and whether developmental or age-related behaviors or physiological processes are targeted.

Model	Genetic Modulation	Deficits	Advantage	Reference
Ts65Dn	Segmental MMU16 trisomy syntenic to Hsa21 + some MMU17	Cognitive loss	Recapitulates DS phenotype	[49]
		Neuronal loss	Most studied model	[17]
		Developmental deficits		
Dp(16)1Yey/+	Triplication of entire MMU16 homologous to Hsa21	Neonatal sleep apnea APP, CTF, Aβ elevated Age-related BFCN loss	More specific for DS More targeted trisomy	[74]
				[75]
				[76]
Ts66Yah	Generated by CRISPR/Cas9	Milder phenotype	Less studied to date May be a more accurate model	[72]
	Excludes the non-Hsa21 genes on MMU16	Learning/memory deficits		[73]
Ts1Cje	Shorter triplication of MMU16 Excludes APP and SOD1	Milder cognitive deficits No BFCN loss	Demonstrates the importance of amyloid	[77]
				[78]
Ts2Cje	Robertsonian translocation MMU16 larger segment than Ts1Cje	BFCN cell loss	Larger segmental trisomy of MMU16 = more severe deficits	[79]
Ts1Rhr	Trisomic for DSCR homologous to Hsa21	Memory deficits	Nearly complete copy of Hsa21q	[80]
Ms1Rhr	Monosomic for DSCR	Memory loss corrected	Important control	[81]
TcMAC21	Mouse Artificial Chr~93% Hsa21 protein genes	Congenital heart defects Learning/memory deficit LPT/neurogenesis delayed	Incomplete studies yet	[82]

## Data Availability

Not applicable.

## Abbreviations

The following abbreviations are used in this manuscript:

MAPT	Microtubule-associated protein tau
AD	Alzheimer’s disease
DS	Down syndrome
DYRK1A	Dual-specificity tyrosine phosphorylation-regulated kinase 1A
RCAN1	Regulator of Calcineurin 1
MMMU	Mouse chromosome
MAC	Mammalian artificial chromosome
Hsa21	Human chromosome 21
ADHD	Attention deficit and hyperactivity disorder
ASD	Autism spectrum disorder
NGF	Nerve growth factor
NFT	Neurofibrillary tangles
DSCR	Down syndrome critical region
